# Magic of high-order van Hove singularity

**DOI:** 10.1038/s41467-019-13670-9

**Published:** 2019-12-18

**Authors:** Noah F. Q. Yuan, Hiroki Isobe, Liang Fu

**Affiliations:** Department of Physics, Massachusetts Institute of Technology, Cambridge, MA 02139 USA

**Keywords:** Two-dimensional materials, Electronic structure, Electronic properties and materials

## Abstract

The van Hove singularity in density of states generally exists in periodic systems due to the presence of saddle points of energy dispersion in momentum space. We introduce a new type of van Hove singularity in two dimensions, resulting from high-order saddle points and exhibiting power-law divergent density of states. We show that high-order van Hove singularity can be generally achieved by tuning the band structure with a single parameter in moiré superlattices, such as twisted bilayer graphene by tuning twist angle or applying pressure, and trilayer graphene by applying vertical electric field. Correlation effects from high-order van Hove singularity near Fermi level are also discussed.

## Abstract (web summary)

Correlation effect is essential to stabilize exotic phases such as superconductivity in twisted bilayer graphene. Here, Yuan et al. predict significantly enhanced electron correlation effects due to an emergent high-order van Hove singularity in a two dimensional moiré superlattice.

## Introduction

The recent discovery of unconventional insulating states and superconductivity in twisted bilayer graphene (TBG)^1,2^ has attracted enormous interest. At ambient pressure, these intriguing phenomena of correlated electrons only occur near a specific twist angle $\theta\,\approx\,1.{1}^{\circ }$, widely referred to as the magic angle. The existence of such a magic angle was first predicted from band structure calculations. Based on a continuum model^3,4^, Bistritzer and MacDonald showed^5^ that the lowest moiré bands in TBG become exceptionally flat at this magic angle, and are hence expected to exhibit strong electron correlation. This pioneering work inspired a large body of experimental works in recent years, which culminated in the discovery reported in refs. ^1,2^. The origin of magic-angle phenomena, i.e., the emergence of superconductivity and correlated insulator, is now under intensive study^6–25^.

While the reduction of bandwidth is undoubtedly important, it is evident that the phenomenology of magic-angle TBG cannot be ascribed to a completely flat band. Quantum oscillation at low magnetic fields reveals doping-dependent Fermi surfaces within the narrow moiré band. Detailed scanning tunneling spectroscopy (STS) studies^26–28^ and compressibility measurements^29^ show that the moiré bandwidth at a magic angle is a few tens of meV, larger than what previous calculations found^5,30–35^. Moreover, there is no direct evidence from existing STS measurements at various twist angles that the moiré band is exceptionally flat right at the magic angle (see also ref. ^28^). These experimental results motivated us to consider additional feature of electronic structure which may create favorable condition for correlated electron phenomena in magic-angle TBG.

One such feature is the van Hove singularity (VHS) in moiré bands. The importance of VHS has already been recognized in our theory of correlated TBG^36,37^ and related studies^38–44^ using the weak coupling approach. Generally speaking, VHS with divergent density of states (DOS) in two-dimensional systems are associated with saddle points of energy dispersion in ${\bf{k}}$ space. When a VHS is close to Fermi energy, the increased DOS amplifies electron correlation, resulting in various ordering instabilities, such as density wave and superconductivity at low temperature^36^. Indeed, the two recent STS measurements on gate-tunable TBG around magic angle^26,27^ find that when the VHS shifts to the Fermi level under gating (the corresponding density is within 10% of half filling), the VHS peak in tunneling density of states splits into two new peaks (see also ref. ^45^). These findings clearly demonstrate the prominent role of VHS in magic-angle TBG. However, it is also known from general consideration and previous STS measurements^46–48^ that VHS are present at all twist angles. It is therefore unclear whether VHS at magic angle is anything special.

In this work, we propose a new perspective that relates magic angle to a new type of VHS in the single-particle energy spectrum of TBG. We show that as the twist angle decreases below a critical value ${\theta }_{c}$, the van Hove saddle point—which marks the change of topology in Fermi surface (Lifshitz transition)—undergoes a topological transition whereby a single saddle point splits into two new ones. Right at ${\theta }_{c}$, the saddle point changes from second-order to higher-order; the DOS at VHS is significantly enhanced from logarithmic to power-law divergence, which promises stronger electron correlation. We propose that proximity to such “high-order van Hove singularity”, which requires tuning to the critical twist angle or pressure, is an important factor responsible for correlated electron phenomena in TBG near half filling.

**Fig. 1:**
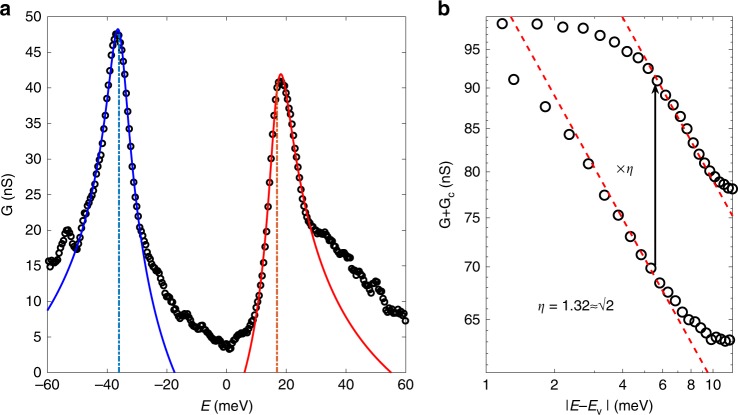
**Theoretical fit to the tunneling conductance measurement**^26^. **a** Open circles are tunneling conductance $G$ of twisted bilayer graphene at twist angle $1.1{0}^{\circ }$, and solid lines are fitting according to Eq. (5) with details given in the Supplementary Material Sec. I. Dashed lines denote singularity energies, indicating the asymmetry of conductance peaks. **b** The peak at ${E}_{{\rm{v}}}\,=\,16.72$ meV plotted in logarithmic scales. Electron and hole sides of the peak fall into two parallel lines with the same slope $-1/4$ and asymmetry ratio $\eta\,=\,1.32\,\approx\,\sqrt{2}$. The only parameter in **b** is the background offset ${G}_{c}\,=\,57.6$ nS.

We demonstrate by topological argument that high-order VHS can be generally achieved by tuning the band structure with just a single control parameter. For TBG, besides twist angle, pressure can also induce superconductivity at a twist angle larger than $1.{1}^{\circ }$^49^. By tracking the evolution of the moiré band with twist angle and pressure, we locate the high-order VHS in experimentally relevant parameter regime and predict its key feature, a distinctive asymmetric peak in DOS. This feature compares well with the experimentally observed VHS peak in magic-angle TBG, as shown in Fig. 1. We also discuss the splitting of VHS peak by nematic or density wave order.

## Results

### Ordinary and high-order VHS

In two-dimensional (2D) electron systems with energy dispersion $E({\bf{k}})$, an ordinary VHS with logarithmically diverging DOS occurs at a saddle point ${{\bf{k}}}_{s}$, determined by

1 $${\nabla }_{{\bf{k}}}E={\bf{0}}\,{\text{and}}\,\det D\;<\;0,$$

where ${D}_{ij}\equiv \frac{1}{2}{\partial }_{i}{\partial }_{j}E$ is the $2\;\times 2$ Hessian matrix of $E$ at ${{\bf{k}}}_{s}$. Since $D$ is symmetric by definition, we can rotate the axes to diagonalize $D$ and Taylor expand the energy dispersion near ${{\bf{k}}}_{s}$ as $E-{E}_{{\rm{v}}}=-\alpha {p}_{x}^{2}+\beta {p}_{y}^{2}$, where ${E}_{{\rm{v}}}$ is the VHS energy, the momentum ${\bf{p}}\,=\,{\bf{k}}\,-\,{{\bf{k}}}_{s}$ is measured from the saddle point, and the coefficients $-\alpha$, $\beta$ are the two eigenvalues of $D$ with $-\alpha \beta =\det D\;<\;0$. This dispersion describes *two* pieces of Fermi contours that intersect at the saddle point ${{\bf{k}}}_{s}$. It is known from a topological consideration that due to the periodicity of $E({\bf{k}})$ on the Brillouin zone (a torus), van Hove saddle points appear quite generally in energy bands of two-dimensional materials^50^.

We now introduce the concept of high-order VHS associated with a high-order saddle point defined by the following condition:

2 $${\nabla }_{{\bf{k}}}E={\bf{0}}\,{\text{and}}\,\det D=0,$$

which imply $\alpha \beta =0$. There exist two types of high-order VHS. The first type corresponds to $\alpha =\beta =0$, i.e., $D={\bf{0}}$. The Taylor expansion of $E({\bf{k}})$ around such saddle point then starts from at least the third order, and describes an intersection of three or more Fermi surfaces at a common ${\bf{k}}$ point^51^. Such high-order VHS (type-I) is also referred to as multicritical VHS in the recent literature^52–54^.

Here we focus instead on a new type of high-order VHS relevant for TBG, where the Hessian matrix $D$ has a single zero eigenvalue ($\beta =0$), while the other eigenvalue is nonzero ($\alpha \;\ne\; 0$). Then in the Taylor expansion of $E({\bf{k}})$, besides the single second-order term $\alpha$ we must also include higher order terms in order to capture the Fermi contours nearby. Importantly, unlike the previous case, type-II high-order VHS still describes the touching of two Fermi surfaces, but they touch tangentially (generally speaking) rather than intersect at a finite angle as in the case of ordinary VHS.

### VHS in TBG

We now turn to VHS in TBG, whose low-energy moiré bands arise from inter-layer coupling of Dirac states on the two graphene layers. Since single-particle scattering between ${\bf{K}}$ and ${\bf{K}}^{\prime}$ points requires large momentum transfer, it is suppressed at small twist angles due to the long wavelength of moiré potential. In the absence of valley hybridization, the moiré bands from ${\bf{K}}$ and ${\bf{K}}^{\prime}$ valleys are decoupled^3–5,55^, with energy dispersions denoted by ${E}_{+}({\bf{k}})$ and ${E}_{-}({\bf{k}})$, respectively. Time-reversal symmetry implies ${E}_{-}({\bf{k}})={E}_{+}(-{\bf{k}})$, so it suffices to consider ${E}_{+}({\bf{k}})$ only in the following.

**Fig. 2:**
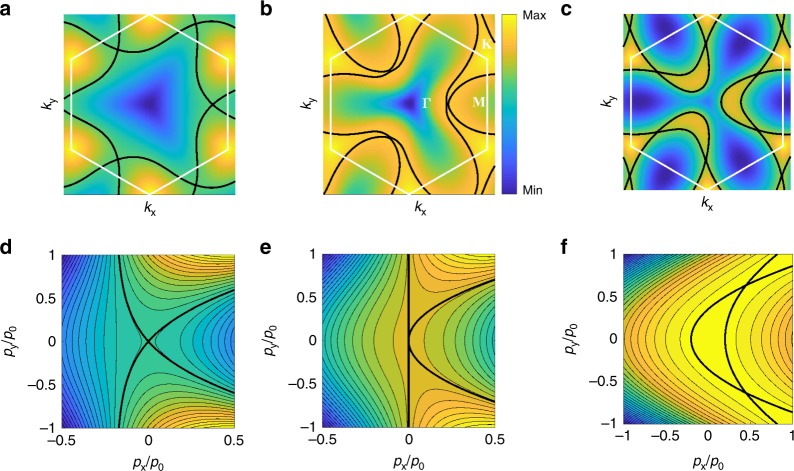
**Energy contours during the topological transition of van Hove singularity (VHS).** Energy contours from band structure calculation are shown in **a**–**c** and from the polynomial expansion around saddle point (3) in **d**–**f**. Thick lines are energy contours at VHS energy. In band structure calculation, $g^{\prime} =1.2\ g$ and the parameters are **a** $g=1$, **b** $g=1.9$, and **c** $g=2$. In **d**–**f**, ${p}_{0}=\alpha /\tilde{\gamma }$ and the parameters are **d** $\beta =0.2\alpha ,\;\kappa =0$, **e** $\beta =0,\;\kappa =0$, and **f** $\beta =-0.2\alpha ,\;\kappa =-0.2{\gamma }^{2}/\alpha$.

The number and location of VHS points in TBG depend on the twist angle. For example, the band structure calculation for $\theta ={2}^{\circ }$^56^ reveals the existence of three symmetry-related van Hove saddle points on $\Gamma M$ lines in the mini-Brillouin zone (MBZ), shown in Fig. 2a. Across the VHS energy, the Fermi contour changes from two disjoint Dirac pockets around MBZ corners to a single pocket enclosing the MBZ center. This leads to the conversion between electron and hole charge carriers, as evidenced by the sign change of Hall coefficient^56^. On the other hand, at $\theta =1.0{5}^{\circ }$, on each $\Gamma M$ line there is a local energy maximum—instead of saddle point—in the moiré valence band. As doping increases, an additional hole pocket (not present at $\theta ={2}^{\circ }$) emerges out of each energy maximum and eventually intersects two Dirac pockets at two new saddle points on opposite sides of $\Gamma M$^32^. In such band structure there are a total of 6 VHS points, shown in Fig. 2c.

By continuity, we deduce from this change in the number of VHS points that a topological transition of saddle points must occur, at a hitherto unknown critical twist angle, in such a way that a saddle point on $\Gamma M$ (denoted by ${\Lambda }_{0}$) splits into a pair of new ones off $\Gamma M$ (denoted by ${\Lambda }_{\pm }$). Importantly, the behavior of these VHS points in the vicinity of this transition is solely governed by the *local* energy dispersion near ${\Lambda }_{0}$.

We now expand $E({\bf{k}})$ near ${\Lambda }_{0}$ to higher orders:

3 $$E-{E}_{{\rm{v}}}=-\alpha {p}_{x}^{2}+\beta {p}_{y}^{2}+\gamma {p}_{x}{p}_{y}^{2}+\kappa {p}_{y}^{4}+\ldots ,$$

where ${p}_{x}$ (${p}_{y}$) is parallel (perpendicular) to $\Gamma M$ line. In the expansion (3), first-order terms vanish because ${\Lambda }_{0}$ has by definition zero Fermi velocity. Moreover, only even power terms of ${p}_{y}$ are allowed because of the two-fold rotation symmetry of TBG, which acts within each valley and maps $({p}_{x},{p}_{y})$ to $({p}_{x},-{p}_{y})$. The third-order $\gamma$ term and fourth-order $\kappa$ term are essential to describe the splitting of VHS across the transition, which we shall show below along with the relation to scaling properties.

The behavior of VHS and Fermi contour of the energy dispersion (3) depends crucially on the sign of $\beta$. For $\beta\;> \; 0$, ${\bf{p}}={\bf{0}}$ (i.e., ${\Lambda }_{0}$) is an ordinary van Hove saddle point with logarithmic divergent DOS. The Fermi contour at the VHS energy consists of two curves that approach straight lines ${p}_{y}/{p}_{x}=\pm\!\sqrt{\alpha /\beta }$ as ${\bf{p}}\to {\bf{0}}$, intersecting at a finite angle $\varphi =2\arctan (\sqrt{\beta /\alpha })$ as shown in Fig. 2d. This behavior corresponds to the VHS in the calculated band structure of TBG at $\theta ={2}^{\circ }$. When $\beta \to {0}^{+}$, $\varphi \to 0$ so that the two Fermi contours at the VHS energy tend to touch tangentially. On the other hand, for $\beta\; <\;0$, ${\bf{p}}={\bf{0}}$ becomes a local energy maximum, while two new saddle points appear at momenta

4 $${\Lambda }_{\pm }=(-\beta \gamma /{\tilde{\gamma }}^{2},\pm\!\sqrt{-2\alpha \beta }/\tilde{\gamma })$$

whose energy is shifted from ${E}_{{\rm{v}}}$ by $\delta =-\alpha {\beta }^{2}/{\tilde{\gamma }}^{2}\;<\;0$, where $\tilde{\gamma }\equiv \sqrt{{\gamma }^{2}+4\alpha \kappa }$. Throughout this manuscript we consider the regime ${\gamma }^{2}+4\alpha \kappa \;> \; 0$ so that $\tilde{\gamma }$ is always real and positive, and two split saddle points ${\Lambda }_{\pm }$ are well-defined. ${\Lambda }_{+}$ and ${\Lambda }_{-}$ are a pair of ordinary VHS points related by two-fold rotation, whose Fermi contours are two parabolas $2\alpha ({p}_{x}\mp \beta /\tilde{\gamma })=(\gamma \pm \tilde{\gamma }){p}_{y}^{2}$, see Fig. 2f. This behavior corresponds to the VHS in the calculated band structure at $\theta =1.0{5}^{\circ }$. In the limit $\beta \to {0}^{-}$, ${\Lambda }_{\pm }$ approaches ${\Lambda }_{0}$.

Our unified description of two regimes of ordinary VHS (${\Lambda }_{0}$ and ${\Lambda }_{\pm }$) using a single local energy dispersion (3) implies that the transition between them corresponds to the sign change of $\beta$. In this process, energy contours at VHS changes from intersecting at one point ${\Lambda }_{0}$ (Fig. 2d and e) to two points ${\Lambda }_{\pm }$ (Fig. 2f). Right at $\beta =0$, ${\bf{p}}={\bf{0}}$ becomes a high-order VHS point. The Fermi contour in its vicinity consists of two parabolas $2\alpha {p}_{x}=(\gamma \pm \tilde{\gamma }){p}_{y}^{2}$, touching each other tangentially at ${\bf{p}}={\bf{0}}$. In the special case with $\kappa =0$, one of the parabolas become a straight line, as shown in Fig. 2e.

The DOS can be used as an indicator of this topological transition of VHS. For the energy dispersion (3), we find the DOS analytically

5 $$\rho (E) 	=\frac{-1}{\pi }{\rm{Im}}\int \frac{{d}^{2}{\bf{p}}}{{(2\pi )}^{2}}\frac{1}{E+i{0}^{+}-E({\bf{p}})}\\ 	=\frac{\,\text{sgn}\,(\alpha \beta )}{\sqrt{2}\alpha {\pi }^{2}}{\rm{Re}}\left[\frac{1}{\sqrt{{z}_{-}}}{\rm{K}}\left(1-\frac{{z}_{+}}{{z}_{-}}\right)-\frac{2i}{\sqrt{{z}_{+}}}{\rm{K}}\left(\frac{{z}_{-}}{{z}_{+}}\right)\Theta (-\alpha \beta )\right],$$

where ${z}_{\pm }=(\beta /\alpha )\pm \sqrt{{(\beta /\alpha )}^{2}+\varepsilon }$ and $\varepsilon ={\tilde{\gamma }}^{2}(E-{E}_{{\rm{v}}})/{\alpha }^{3}$ is energy deviation from VHS in dimensionless unit. The integration over ${\bf{p}}$ is extended to infinity. Here ${\rm{K}}(z)$ is the complete elliptic integral of the first kind, ${\rm{sgn}}(r)$ is the sign function defined as ${\rm{sgn}}(r)=-1$ for $r\;<\;0$ and $1$ for $r\ge 0$, and $\Theta (r)\equiv \frac{1}{2}[1-{\rm{sgn}}(-r)]$ is the step function so that $\Theta (0)=0$.

**Fig. 3:**
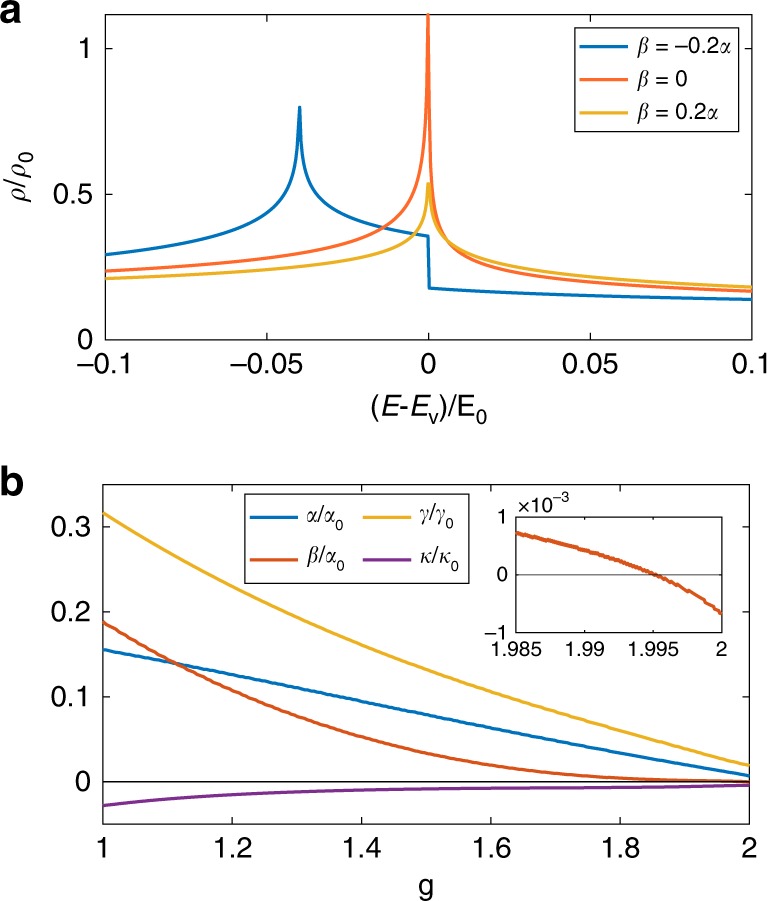
**Density of states (DOS) and coefficients of Taylor expansion around saddle point during the topological transition of VHS.** **a** DOS of Eq. (5) with different $\beta$, where ${E}_{0}={\alpha }^{3}/{\tilde{\gamma }}^{2},\;{\rho }_{0}={\alpha }^{-1}$. **b** Normalized coefficients of Eq. (3) as functions of $g$ when $g^{\prime} =1.2\ g$. Here ${\alpha }_{0}=\lambda v,{\gamma }_{0}={\lambda }^{2}v$ and ${\kappa }_{0}={\lambda }^{3}\ v$. Inset of **b** is zoom-in plot where $\beta$ changes sign near high-order VHS while $\alpha ,\gamma ,\kappa$ do not.

The DOS with different $\beta$ in Eq. (5) are shown in Fig. 3a. In the following we will discuss the asymptotic behavior of the DOS near ordinary and high-order VHS. When $\alpha \;> \; 0$ and $\beta \;\ne\; 0$, the DOS diverges logarithmically at ordinary VHS:

6 $$\rho (E)=\frac{1}{4{\pi }^{2}\sqrt{\alpha | \beta | }}\times \left\{\begin{array}{ll}{\log}\frac{\Lambda }{| E-{E}_{{\rm{v}}}| }&\beta \, {> } \, 0\\ \sqrt{2}{\log}\frac{\Lambda }{| E-{E}_{{\rm{v}}}-\delta | }&\beta \, < \, 0\end{array}\right.$$

where $\Lambda =64\alpha {\beta }^{2}/{\tilde{\gamma }}^{2}$ is the high-energy cutoff. In the limit $\beta \to 0$, the prefactor in Eq. (6) diverges as $1/\sqrt{| \beta | }$, indicating a strong increase of DOS as the VHS approaches a high-order one. Right at the transition point $\beta =0$, we find power-law divergent DOS near high-order VHS

7 $$\rho (E)=\frac{C}{{\root{4}\of{4\alpha {\tilde{\gamma }}^{2}}}}\times \left\{\begin{array}{ll}{(E-{E}_{{\rm{v}}})}^{-\frac{1}{4}}&E\,{> }\, {E}_{{\rm{v}}}\\ \sqrt{2}{({E}_{{\rm{v}}}-E)}^{-\frac{1}{4}}&E\,{<}\,{E}_{{\rm{v}}}\end{array}\right.$$

where $C={(2\pi )}^{-\frac{5}{2}}\Gamma {(\frac{1}{4})}^{2}=0.133$. This power-law divergence with exponent $-1/4$ is stronger than the logarithmic divergence at ordinary VHS. Another key difference is that the diverging part of DOS around this high-order VHS is inherently asymmetric with respect to ${E}_{{\rm{v}}}$, as reflected by the $\sqrt{2}$ factor in Eq. (7). This is in contrast with ordinary VHS where the DOS above and below are asymptotically symmetric. Using Eq. (5), we can fit the experimental data of tunneling conductance in ref. ^26^ with finite broadening, as shown in Fig. 1a and Supplementary Material Sec. I.

At the high-order VHS ($\beta =0$), the dispersion has the following scaling invariance

8 $$E({\lambda }^{\frac{1}{2}}{p}_{x},{\lambda }^{\frac{1}{4}}{p}_{y})=\lambda E({p}_{x},{p}_{y})$$

with arbitrary $\lambda \;> \; 0$. Here we set ${E}_{{\rm{v}}}=0$ for simplicity. Including higher order terms or the third and fourth order terms other than $\gamma ,\kappa$ in Taylor expansion (3) will break scaling invariance (8). Notice that the scaling dimensions along ${p}_{x}$ and ${p}_{y}$ directions are different $[{p}_{x}]=\frac{1}{2},[{p}_{y}]=\frac{1}{4}$, with scaling dimension of energy $[E]=1$. From the scaling invariance (8) we immediately obtain the scaling dimension of DOS as $[\rho ]=[{p}_{x}]+[{p}_{y}]-[E]=-\frac{1}{4}$, the same as Eq. (7). To directly reveal the power-law behavior of experimental data, in Fig. 1b we plot energy deviation $| E-{E}_{{\rm{v}}}|$ and experimental DOS including background contribution both in logarithmic scale. In the log-log plot, two sides of the high-order VHS peak form two parallel lines with the same slope $-1/4$, and the particle-hole asymmetry ratio is 1.32 close to $\sqrt{2}$, which are both consistent with Eq. (7).

Besides $\alpha$ and $\beta$, the Fermi contours in momentum space depend on both $\gamma$ and $\kappa$, while the DOS in energy domain depends only on $\tilde{\gamma }$. This is because the same scaling dimension of ${p}_{x}$ and ${p}_{y}^{2}$ allows the nonlinear transform

9 $${\tilde{p}}_{x}={p}_{x}-\frac{\gamma }{2\alpha }{p}_{y}^{2},\quad {\tilde{p}}_{y}={p}_{y},$$

under which the dispersion (3) becomes $E-{E}_{{\rm{v}}}=-\alpha {\tilde{p}}_{x}^{2}+\beta {\tilde{p}}_{y}^{2}+{\tilde{\gamma }}^{2}{\tilde{p}}_{y}^{4}/(4\alpha )$. The nonlinear transform (9) changes the shape of Fermi contours while it preserves area element and hence leaves DOS in Eq. (5) invariant.

We have thus established by continuity argument the existence of high-order VHS in the moiré band of TBG at a certain critical twist angle ${\theta }_{c}$. The value of ${\theta }_{c}$ depends on the model we employ. As an illustrative example, we consider the continuum model^3–5,32^ of TBG, where approximate particle-hole symmetry holds, and the Fermi surface topology is solely determined by two dimensionless parameters $g=\lambda u/v,g^{\prime} =\lambda u^{\prime} /v$. Here $u$ and $u^{\prime}$ denote interlayer hoppings in AA and AB regions respectively, $v$ is the monolayer Dirac velocity and $\lambda$ is the moiré wavelength. We further fix the ratio $g^{\prime} /g=u^{\prime} /u=1.2$ as calculated for corrugated structure at $\theta \sim {1}^{\circ }$^32^. At given $g\in [1,2]$, we calculate the Taylor coefficients $\alpha ,\beta ,\gamma ,\kappa$ by numerical derivatives of energy with respect to momentum at VHS on the hole side, and the result is shown in Fig. 3b. It can be found that ${\gamma }^{2}+4\alpha \kappa$ is always positive when $g\in [1,2]$, and across ${g}_{c}\approx 1.995$, $\beta$ changes sign (Fig. 3b inset) while $\alpha ,\gamma ,\kappa$ do not. With appropriate and reasonable values of $u$ and $v$, from ${g}_{c}$ we can obtain the critical twist angle ${\theta }_{c}\approx {1}^{\circ }$, consistent with experiments. Details of continuum model can be found in the Supplementary Material Sec. II. We may include more ingredients such as lattice relaxation^31,57,58^ and strain^59^ to better describe TBG, thus the Fermi surface topology will depend on more details of the model. Nevertheless, the existence of high-order VHS and critical twist angle ${\theta }_{c}$ is robust^59^.

### Many-body phenomena near high-order VHS

When chemical potential is near high-order VHS, many-body effects can be drastic as the strongly diverging DOS leads to divergences in noninteracting susceptibilities of various channels. This signals a strong tendency to various broken symmetry states around van Hove filling, when electron-electron interaction is taken into account. Indeed, the recent STS measurements found that when the Fermi energy approaches the van Hove energy under doping, the VHS peak in DOS splits into two new ones^26,27^.

A detailed analysis of interacting electrons near high-order VHS is beyond the scope of this work. Instead we discuss two possible scenarios for the observed splitting of the VHS peak near the Fermi energy.

First, strains in experimental samples can split DOS peak by breaking rotation symmetries which relate VHS along different directions. Though the energy splitting due to strain may be small at single-particle level, Coulomb interaction $U$ can give rise to the Stoner-type enhancement factor ${(1-U{\chi }_{s})}^{-1}$ within the random phase approximation (RPA). Since the noninteracting susceptibility ${\chi }_{s}$ reflects the divergent DOS, strain effect on high-order VHS can be greatly enhanced by interaction.

**Fig. 4:**
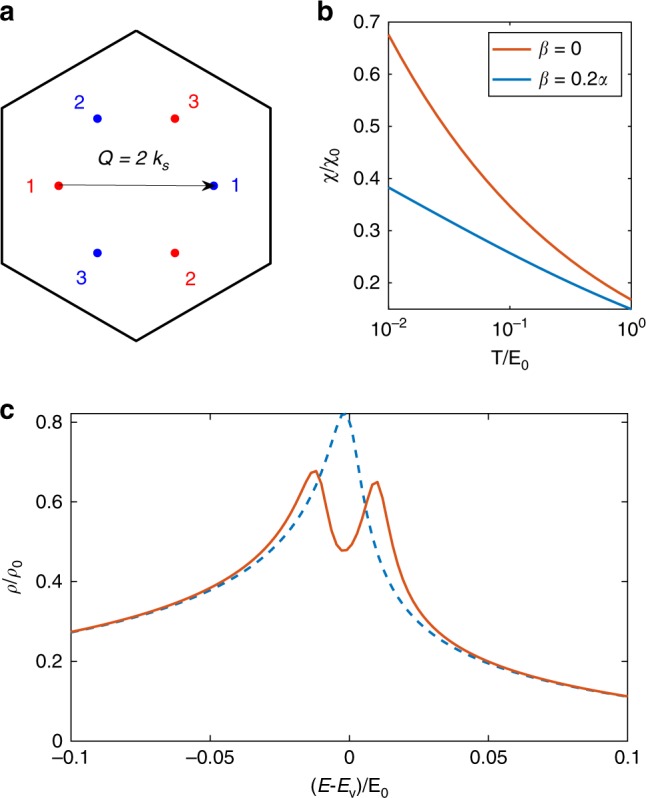
**Particle-hole nesting and density wave instability in twisted bilayer graphene.** **a** Six high-order VHS in MBZ, where different colors denote different valleys, and 1, 2, 3 denote three $\Gamma M$ directions. **b** Temperature dependence of intervalley susceptibility $\chi \equiv \chi ({\bf{Q}},{E}_{\text{v}})$ at ordinary ($\beta =0.2\alpha$) and high-order ($\beta =0$) VHS with ${\bf{Q}}=2{{\bf{k}}}_{s}$ and ${\chi }_{0}={\alpha }^{-1}$. **c** Density of states with (solid) and without (dashed) intervalley density wave. Here ${E}_{0}={\alpha }^{3}/{\tilde{\gamma }}^{2}$ and details can be found in the Methods section.

Second, an intervalley density wave order can split a DOS peak by spontaneously breaking translation symmetry. To show such a density wave instability, we calculate intervalley susceptibility $\chi ({\bf{q}})$ with finite momentum ${\bf{q}}$. There are in total six high-order VHS from two valleys, located at three $\Gamma M$ lines (Fig. 4a). When we consider two high-order VHS along the same $\Gamma M$ line but from opposite valleys, $\chi ({\bf{q}})$ is sharply enhanced near wave vector ${\bf{q}}={\bf{Q}}$ due to the power-law divergent DOS from both valleys, where ${\bf{Q}}=2{{\bf{k}}}_{s}$ is the momentum separation between the two VHS (Fig. 4a). As a result, when approaching low temperatures, the intervalley susceptibility $\chi ({\bf{Q}})$ of high-order VHS diverges more rapidly than logarithm (Fig. 4b), indicating a stronger tendency toward density wave instability than one with ordinary VHS. The DOS peak at VHS energy ${E}_{{\rm{v}}}$ is split into two in the presence of intervalley density wave (Fig. 4c).

## Discussion

To summarize, we propose that the proximity to high-order VHS underlies correlated electron phenomena in TBG near magic angle. We reveal a distinctive feature of high-order VHS—power-law divergent DOS with an asymmetric peak, which compares well with the recent STS data on magic-angle TBG. We also discuss nematic and density wave instabilities due to electron-electron interaction near van Hove filling, and illustrate the splitting of VHS in these broken symmetry states.

It is worth examining the similarity and difference between the two scenarios favoring correlated electron phenomena in TBG: flat band^5^ and (high-order) VHS. Both scenarios rely on large DOS enhancing electron correlation. Obviously, the largest possible DOS is realized in the limit of a completely flat band^60^. However, a completely flat band is difficult to achieve under realistic conditions. On the other hand, the VHS scenario is more likely to be relevant when the bandwidth is not so small in comparison to electron interaction. In this scenario, the DOS near Fermi energy matters more than those far away, and it is further increased by proximity to high-order VHS. Importantly, to achieve high-order VHS generally requires tuning the band structure with only a single parameter, whereas to achieve a completely or extremely flat band usually requires more fine tuning.

Broadly speaking, moiré superlattices in 2D materials offer an unprecedented platform for studying correlated electron phenomena near VHS. Electrostatic gating enables doping these systems to van Hove filling without introducing disorder. The great tunability of moiré band structure by twist angle, electric field, strain and other means grants access to high-order VHS, where the strongly divergent DOS promises a plethora of many-body phenomena. Such examples include TBG discussed in the maintext and trilayer graphene on boron nitride discussed in Supplementary Material Sec. III. The time is coming for creating strong electron correlation by designing single-particle dispersion around VHS.

## Methods

### Fitting of tunneling conductance peaks

In Eq. (5) we assume the dispersion (3) extends to the whole momentum space, which holds only for a finite momentum range in realistic systems. Assuming the dispersion over the whole momentum space as ${{\mathcal{E}}}_{{\bf{p}}}$, whose Taylor expansion is ${E}_{{\bf{p}}}$ in the finite momentum range $| {\bf{p}}| \;<\;\Lambda$, we find the total DOS of such dispersion can be written as

10 $${\rho }_{{\rm{tot}}}(E)=\int \frac{{d}^{2}{\bf{p}}}{{(2\pi )}^{2}}\delta (E-{{\mathcal{E}}}_{{\bf{p}}})=\rho (E)+{\rho }_{c},$$

where $\rho (E)$ is Eq. (5) if the Taylor expansion is Eq. (5), and ${\rho }_{c}$ is the background contribution

11 $${\rho }_{c}={\int }_{| {\bf{p}}| > \Lambda }\frac{{d}^{2}{\bf{p}}}{{(2\pi )}^{2}}[\delta (E-{{\mathcal{E}}}_{{\bf{p}}})-\delta (E-{E}_{{\bf{p}}})].$$

When DOS of the tip is featureless, the tunneling conductance from the tip to the sample will be proportional to the total DOS ${\rho }_{{\rm{tot}}}$ of the sample. There are two distinct types of divergent DOS and hence conductance peaks in this work and also in experimental data. The first type is due to ordinary VHS, which is symmetric and logarithmic, and another type is due to high-order VHS, which is intrinsically asymmetric and power-law. These two types of conductance peaks follow different functional forms as follows ($o$ denotes ordinary VHS and $h$ denotes high-order VHS)

12 $${G}_{o}(E)=A\times \mathrm{log}| E-{E}_{{\rm{v}}}| -{G}_{c},$$

13 $${G}_{h}(E)=A\times {\rm{Re}}{({E}_{{\rm{v}}}-E)}^{-\frac{1}{4}}-{G}_{c},$$

each with three parameters $A,{E}_{{\rm{v}}}$ and ${G}_{c}$. Among them, ${E}_{{\rm{v}}}$ denotes the energy of conductance peak, and ${G}_{c}$ is the background contribution.

In Fig. 1b, we plot conductance data $G$ near the peaks at twist angle $1.1{0}^{\circ }$, where energy and conductance are both plot in logarithmic scale. Since the VHS is high-order, conductance peak is asymmetric and power-law, two sides of the peak will follow two parallel lines with the same slope −1/4 but different vertical intercepts.

We make the substitution $E\to E+i\eta$ in the expressions above of tunneling conductance to describe broadening effect. The two sides of the DOS peak of ordinary VHS are broadened in the same way, while the two sides of the DOS peak of high-order VHS are broadened differently due to the intrinsic particle-hole asymmetry. As a result, after broadening, ${E}_{{\rm{v}}}$ coincides with the DOS peak energy ${E}_{{\rm{m}}}$ when VHS is ordinary, while for high-order VHS, ${E}_{{\rm{v}}}$ can deviate from ${E}_{{\rm{m}}}$ as shown in Fig. 1a.

### Mean-field Hamiltonian of interacting high-order VHS

With finite intervalley density wave order parameter $\Delta$, the low-energy Hamiltonian of two high-order VHS on the same $\Gamma M$ line can be written as ${\mathcal{H}}({\bf{p}})={E}_{{\rm{v}}}-\alpha {p}_{x}^{2}+\kappa {p}_{y}^{4}+\gamma {p}_{x}{p}_{y}^{2}{\tau }_{z}+\Delta {\tau }_{x},$ where Pauli matrices ${\boldsymbol{\tau }}$ act on the valley indices. From this Hamiltonian Fig. 4c is plotted, with order parameter $\Delta =0.01{E}_{0}$ and broadening $\eta =5\;\times 1{0}^{-3}{E}_{0}$.

## Supplementary information

Supplementary Information

## Data Availability

The data that support the findings of this study are available from the corresponding author upon reasonable request.
