# Supplementary material for: Magic of high-order van Hove singularity
Source: Nat Commun. 2019 Dec 18;10:5769. doi: 10.1038/s41467-019-13670-9 (PMC6920381; doi:10.1038/s41467-019-13670-9)
Supplement: Supplementary file 1 — Supplementary Information [file 41467_2019_13670_MOESM1_ESM.pdf]

Supplemental Material for "Magic of high-order van Hove singularity"

Noah F. Q. Yuan, Hiroki Isobe, and Liang Fu
Department of Physics, Massachusetts Institute of Technology, Cambridge, Massachusetts 02139, USA

I. SUPPLEMENTARY METHODS: THEORETICAL FITTING OF TUNNELING CONDUCTANCE PEAKS

The dispersion near a van Hove singularity (VHS) is

$$E - E_{\rm v} = -\alpha p_x^2 + \beta p_y^2 + \gamma p_x p_y^2 + \kappa p_y^4, \tag{1}$$

and the corresponding density of states (DOS) is

$$\rho(E) = \frac{1}{\sqrt{2}\alpha\pi^2} Q(\varepsilon, r), \quad \varepsilon = \frac{\tilde{\gamma}^2}{\alpha^3}(E + i\eta - E_{\rm v}), \quad r = \frac{\beta}{\alpha}, \tag{2}$$

where $Q \equiv \text{sgn}(r)\,[\text{Re}f + \Theta(-r)\text{Im}g]$, $\tilde{\gamma} = \sqrt{\gamma^2 + 4\alpha\kappa}$ and

$$f(\varepsilon, r) = \frac{1}{\sqrt{z_-}}\text{K}\left(1 - \frac{z_+}{z_-}\right), \quad g(\varepsilon, r) = \frac{2}{\sqrt{z_+}}\text{K}\left(\frac{z_-}{z_+}\right) \tag{3}$$

with $z_\pm = r \pm \sqrt{r^2 + \varepsilon}$. The elliptic integral we use is defined as

$$\text{K}(z) = \int_0^{\pi/2} \frac{d\theta}{\sqrt{1 - z\sin^2\theta}}. \tag{4}$$

In Eq. (2) we assume the dispersion (1) extends to the whole momentum space, while for realistic systems Eq. (1) holds only for a finite range $|E - E_{\rm v}| < \Omega$. To account for the high-energy cutoff Ω, we introduce a negative background to the DOS expression (2), and hence tunneling conductance becomes

$$G(E) = G_0 Q(\varepsilon, r) - G_c, \tag{5}$$

with two additional parameters G_0 and G_c. Here G_0 is due to the tunneling matrix element between the sample and the tip in STS experiments, and G_c is due to the high-energy cutoff Ω. Notice that Eq. (5) only applies to energy range $|E - E_{\rm v}| < \Omega$.

In total we have 6 parameters $G_0, G_c, E_{\rm v}, \eta, r, E_0 \equiv \alpha^3/\tilde{\gamma}^2$ in the fitting of tunneling conductance. However, due to the scaling property $Q(\lambda^2\varepsilon, \lambda r) = \lambda^{-\frac{1}{2}}Q(\varepsilon, r)$, there are only 5 independent parameters $G_0, G_c, E_{\rm v}, \eta$ and $w \equiv E_0 r^2 \text{sgn}(r) = \alpha\beta^2\text{sgn}(\beta)/\tilde{\gamma}^2$. Here $|w|$ is the low-energy cutoff of the high-order VHS, namely in energy range $|w| < |E - E_{\rm v}| < \Omega$ the VHS can be treated approximately high-order. When $w = 0$ the VHS is exactly high-order.

The optimal fitting parameters in least squares fitting are given below in Table. I for different samples, and fitting results are shown in Fig. 1. We can treat w as the single indicator of the topological transition of VHS similar to β in Eq. (1). From Table. I, it can be found

$$w(2.02^\circ) \gg w(1.10^\circ) \approx 0 > w(0.79^\circ), \tag{6}$$

indicating a topological transition of VHS.

II. SUPPLEMENTARY METHODS: CONTINUUM MODEL

To determine the value of θ_c, we calculate the moiré band structure and track its evolution with twist angle. It is important to note that our calculation of TBG band structure is only to illustrate the Lifshitz transition of VHS and existence of high-order VHS in TBG, thus many ingredients such as strain and in-plane lattice relaxation are neglected.

Our calculation uses a generalized continuum model [2]

$$H(\boldsymbol{k}, \boldsymbol{r}) = \begin{pmatrix} vR_{\theta/2}\boldsymbol{p}_1 \cdot \boldsymbol{\sigma} & U(\boldsymbol{r}) \\ U^\dagger(\boldsymbol{r}) & vR_{-\theta/2}\boldsymbol{p}_2 \cdot \boldsymbol{\sigma} \end{pmatrix}, \quad U(\boldsymbol{r}) = \sum_{j=0}^{2} \begin{pmatrix} u & u'\omega^{-j} \\ u'\omega^j & u \end{pmatrix} e^{i\boldsymbol{G}_j \cdot \boldsymbol{r}}, \tag{7}$$

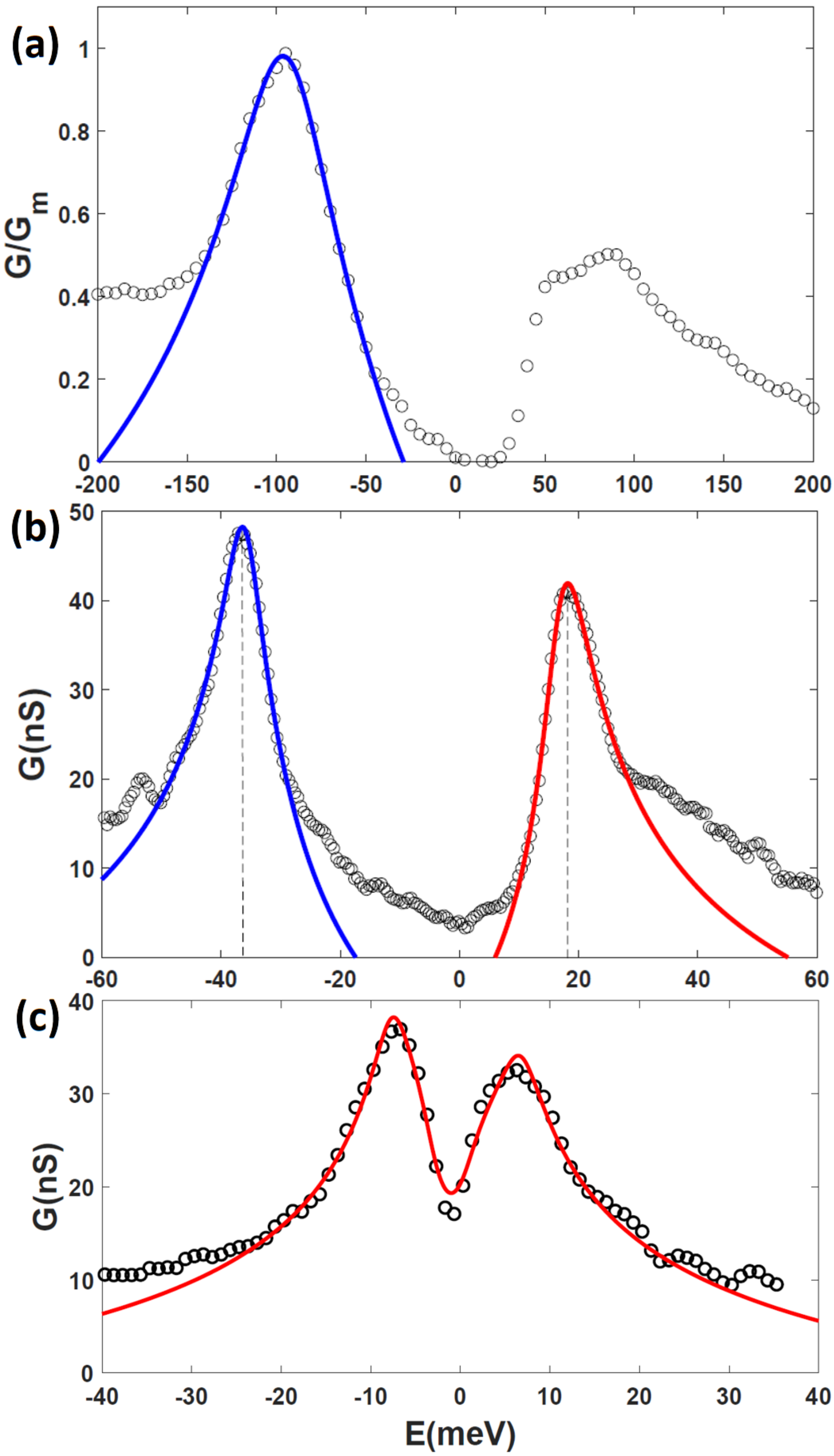

Supplementary Figure 1: Open circles are tunneling conductance data G of twisted bilayer graphene [1] at twist angles (a) 2.02°, (b) 1.10° and (c) 0.79° respectively. Solid lines are Eq. (5) with parameters in Table. I. In (a), $G_{\rm m}$ denotes the maximal value of tunneling conductance data. Dashed lines in (b) denote positions of DOS peak energy, indicating the asymmetry of DOS peaks.

θ	G_0 (nS)	G_c (nS)	$E_{\rm v}$ (meV)	η (meV)	w (meV)	band
2.02°	$6.112G_{\rm m}$	$3.108G_{\rm m}$	-94.15	23.29	49.69	−
1.10°	98.22	67.57	-36.05	2.786	3.360	−
	76.34	57.6	16.72	3.556	-0.1442	+
0.79°	30.89	16.22	-3.299	1.822	-4.271	−
	32.82	16.60	0.7944	2.074	-5.8787	+

Supplementary Table I: Fitting parameters of tunneling conductance data at twist angle θ. In the last column $\pm$ denote positive/negative energy band respectively. For $\theta = 2.02°$, $G_{\rm m}$ denotes the maximal value of tunneling conductance data.

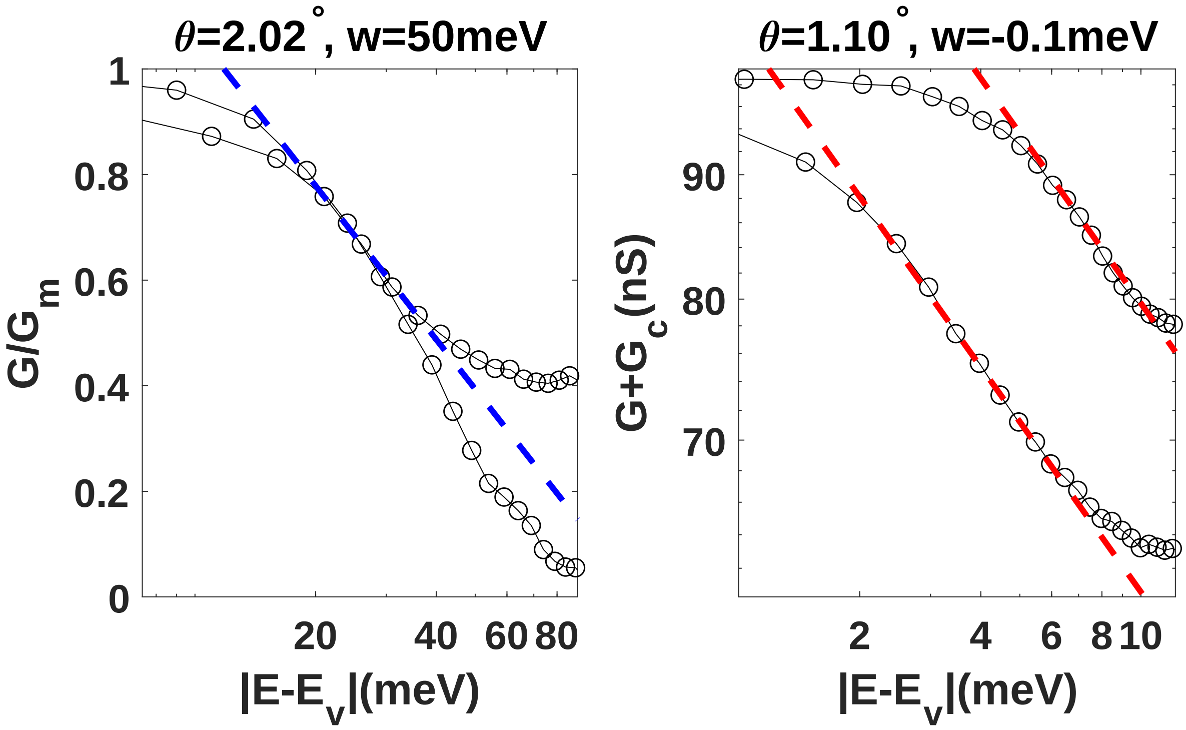

Supplementary Figure 2: Conductance peaks induced by ordinary (left panel) and high-order (right panel) VHS. In both figures, energy is measured from conductance peak energy $E_{\rm v}$ and plot in logarithmic scale, open circles are tunneling conductance data G, and two lines denote two sides of the conductance peak. In the left panel $\theta = 2.02°$, the conductance peak is at negative energy $E_{\rm v} = -94.15$meV < 0, and two sides of the peak collapse to the same dashed blue line when $\eta < |E - E_{\rm v}| < |w|$. In the right panel $\theta = 1.10°$, the conductance peak is at positive energy $E_{\rm v} = 16.72$meV > 0, conductance is in logarithmic scale and two sides of the peak follow two parallel lines when $|E - E_{\rm v}| \gg |w|$. The two dashed red lines have the same slope $-1/4$, and the difference between vertical intercepts is 0.28, deviating from the theoretical value $\log\sqrt{2} = 0.35$ caused by imperfect high-order VHS and finite broadening.

with $\omega = e^{2\pi i/3}$. Here $\boldsymbol{\sigma}$ acts on the two components of massless Dirac fermion at $\boldsymbol{K}$ point of each layer. Due to the relative orientation of the two layers, the Dirac spinors on the two layers are rotated by $\pm\theta/2$ respectively under the orthogonal rotation matrix $R_{\pm\theta/2}$. $\boldsymbol{p}_l = \boldsymbol{k} - \boldsymbol{K}_l$ denotes momentum deviation from the $\boldsymbol{K}$ point in layer $l = 1, 2$. The 2×2 matrix $U(\boldsymbol{r})$ denotes interlayer tunneling and is periodic in real space $U(\boldsymbol{r} + \lambda \boldsymbol{e}_\pm) = U(\boldsymbol{r})$, where $\lambda \boldsymbol{e}_\pm$ are two primitive vectors of TBG moiré superlattice, and $\lambda = a/(2\sin\frac{\theta}{2})$ is the moiré wavelength at twist angle θ with graphene lattice constant a. $U(\boldsymbol{r})$ contains two independent parameters u and u' associated with interlayer tunneling within the same sublattice and between the two sublattices respectively. Due to the out-of-plane lattice relaxation, AA regions have larger interlayer distance than AB regions [1], resulting in $u' < u$ in improved continuum model of TBG band structure [2].

At small θ, Fermi surface topology and hence VHS in the continuum model (7) are solely determined by two dimensionless parameters $g \equiv \lambda u/v$ and $g' \equiv \lambda u'/v$,where v is the Dirac velocity and λ is the moiré wavelength. This

can be seen by following scaling relation

$$H(\lambda^{-1}\boldsymbol{k}, \lambda \boldsymbol{r}) = \frac{v}{\lambda}\begin{pmatrix} \boldsymbol{p}_1 \cdot \boldsymbol{\sigma} & f(\boldsymbol{r}) \\ f^\dagger(\boldsymbol{r}) & \boldsymbol{p}_2 \cdot \boldsymbol{\sigma} \end{pmatrix}, \quad f(\boldsymbol{r}) = \sum_{j=0}^{2} \begin{pmatrix} g & g'\omega^{-j} \\ g'\omega^{j} & g \end{pmatrix} e^{i\boldsymbol{n}_j \cdot \boldsymbol{r}}, \quad \boldsymbol{n}_0 = \boldsymbol{0}, \quad \boldsymbol{n}_{1,2} = (\mp\frac{1}{2}, \frac{\sqrt{3}}{2}). \tag{8}$$

Dimensionless parameters g, g' increase with the moiré wavelength as θ decreases; they also increase with interlayer tunneling under pressure. For simplicity, in the following we fix the ratio $g'/g = u'/u = 1.2$ as calculated for relaxed structure at $\theta \sim 1^\circ$ [2] and study VHS evolution with the single parameter g. Since electron and hole sides are approximately symmetric, we focus on VHS in the hole side.

At $g = 1$ there are three ordinary VHS on ΓM (Fig. 2a of maintext), while at $g = 2$ there are six ordinary VHS on two sides of ΓM (Fig. 2c of maintext). According to our analysis above, this implies a topological transition of VHS occurs at $g = g_c \in (1, 2)$. To determine the value g_c, we calculate the Taylor coefficients $\alpha, \beta, \gamma, \kappa$ by numerical derivatives of energy dispersion with respect to momentum components k_x, k_y at VHS point. As shown in Fig. 3b, $\alpha, \beta, \gamma, |\kappa|$ all decrease as g increases, and across $g_c \approx 1.995$, β changes sign (Fig. 3b inset) while α, γ stay positive and κ stays negative. It can be verified that through the whole process $\gamma^2 + 4\alpha\kappa$ is positive so that the asymptotic behavior is always saddle point.

At $g < g_c$, we find that over an extended parameter range $g \in [1.6, 1.8]$, the van Hove filling is close to two electrons per unit cell (where correlated electron phenomena are observed), in agreement with the observed sign change of Hall coefficient under doping and pressure [3]. Importantly, in this range $0 < \beta \ll \alpha$ and VHS can still be treated approximately as high-order. At small $g - g_c > 0$, the bandwidth decreases rapidly; the band structure calculated from continuum model becomes rather complex above g_c [7]. It is possible that additional effects not captured in Eq. (7), such as in-plane lattice distortion, become important in this regime [4–6]. Nonetheless, the existence of topological transition of VHS as a function of θ is a robust feature.

To translate the parameter g into the actual twist angle requires the knowledge of Dirac velocity v and interlayer hoppings u, u', which are subject to some uncertainty. It is known that Coulomb interaction increases the Dirac velocity substantially at low energy [8–10]. It is recently proposed that this increased velocity may account for the discrepancy between the moiré bandwidth found in STS measurements and in previous calculations [1]. When we use an increased Dirac velocity $va^{-1} = 2.41$eV, 13% larger than DFT value and adopt the interlayer hopping parameters $u = 79.7$meV, $u' = 97.5$meV from Ref. [2], we find $\theta_c = 0.95^\circ$, and the experimentally established range of magic angle $\theta_{\rm exp} \in [1^\circ, 1.2^\circ]$ corresponds to the range $g_{\rm exp} \in [1.6, 1.9]$ where $0 < \beta \ll \alpha$ and VHS is approximately high-order. We can also apply pressure to change interlayer couplings u, u' and hence g, g'. In this way, high-order VHS can be achieved at larger twist angles.

We can also use continuum model instaed of effective model (1) to compute the total density of states. In Fig. 4 we plot the Fermi contours and corresponding total density of states near van Hove singularity in continuum model with different coupling constant g.

In the maintext and also previous sections, we calculate the total density of states which is the averaged DOS over the whole sample. In this section we show that at magic angle, even local density of states (LDOS) also exhibits behaviors of high-order VHS, both in AA and AB regions.

In Fig. 5, by continuum model we calculate LDOS in AA and AB regions of TBG at magic angle as functions of energy, where both LDOS and energy are plot in log scale and $E_{\rm v}$ denotes the energy of conductance peak. Red and blue colors denote peaks at positive and negative energy sides respectively. We find that LDOS in both AA and AB regions show asymmetric power-law divergent peaks, while LDOS in AA region is much larger than LDOS in AB region, which is consistent with experimental data in Ref. [1].

III. SUPPLEMENTARY METHODS: TRILAYER GRAPHENE ON BORON NITRIDE

In trilayer graphene with ABC stacking (ABC-TLG), each two layers form AB stacking, and the geometrical structure is shown in Fig. 6a and b. We denote the three layers as layer 1, 2 and 3, and choose the hexagon center of layer 2 as the three-fold rotation center O. The rotation center is also the registered site of A_1 and B_3 carbon atoms. The axes of coordinate system is also shown in Fig. 6a and b.

Around the origin O, we find three symmetries: The three-fold rotation C_{3z}, the two-fold rotation C_{2x} and mirror reflection $M_x : (x, y, z) \to (-x, y, z)$, which generate the point group D_{3d}.

The low-energy physics of ABC-TLG is described by states at A_1 and B_3 sites near $\pm\boldsymbol{K}$ points of Brillouin zone shown in Fig. 6c. The point group at $\boldsymbol{K}$ point is D_3 with two generators C_{3z} and C_{2x}. Since A_1 and B_3 are at rotation centers of C_{3z}, Bloch waves with momentum $\boldsymbol{K}$ at A_1 and B_3 will carry zero angular momentum under C_{3z} and furnish 1D representations A_1 and A_2 of D_3. As a result, the band spectrum at $\boldsymbol{K}$ point is gapped. In the basis

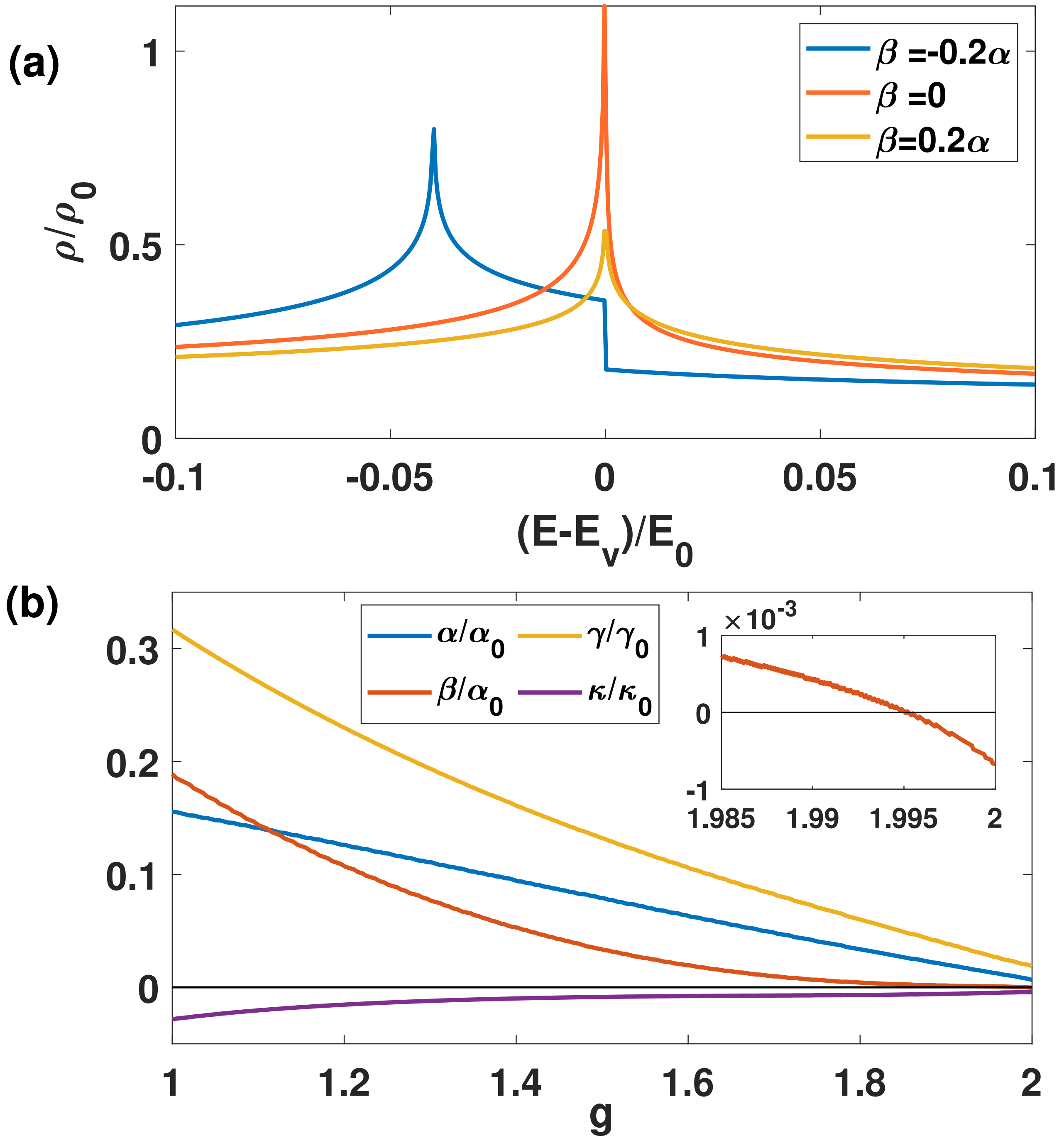

Supplementary Figure 3: (a) DOS of Eq. (1) with different β, where $E_0 = \alpha^3/\tilde{\gamma}^2, \rho_0 = \alpha^{-1}$. (b) Normalized Taylor coefficients of Eq. (1) as functions of g when $g' = 1.2g$. Here $\alpha_0 = \lambda v, \gamma_0 = \lambda^2 v$ and $\kappa_0 = \lambda^3 v$. Inset of (b) is zoom-in plot where β changes sign near high-order VHS while α, γ, κ do not.

of A_1 and B_3, the 2 by 2 effective $k \cdot p$ Hamiltonian near $+\boldsymbol{K}$ point reads [13–15]

$$H(\boldsymbol{p}) = -\mu + ap^2 + (\gamma + bp^2)\sigma_x + c\begin{pmatrix} 0 & p_-^3 \\ p_+^3 & 0 \end{pmatrix} \tag{9}$$

up to the third order in $\boldsymbol{p}$, where $p_\pm = p_x + ip_y$ and $p = |\boldsymbol{p}| = |p_\pm|$. Here the parameters are (unit: eV)

$$\mu = 0.0027, \quad a = 3.2708, \quad \gamma = -0.0083, \quad b = 3.2165, \quad c = 91.1443. \tag{10}$$

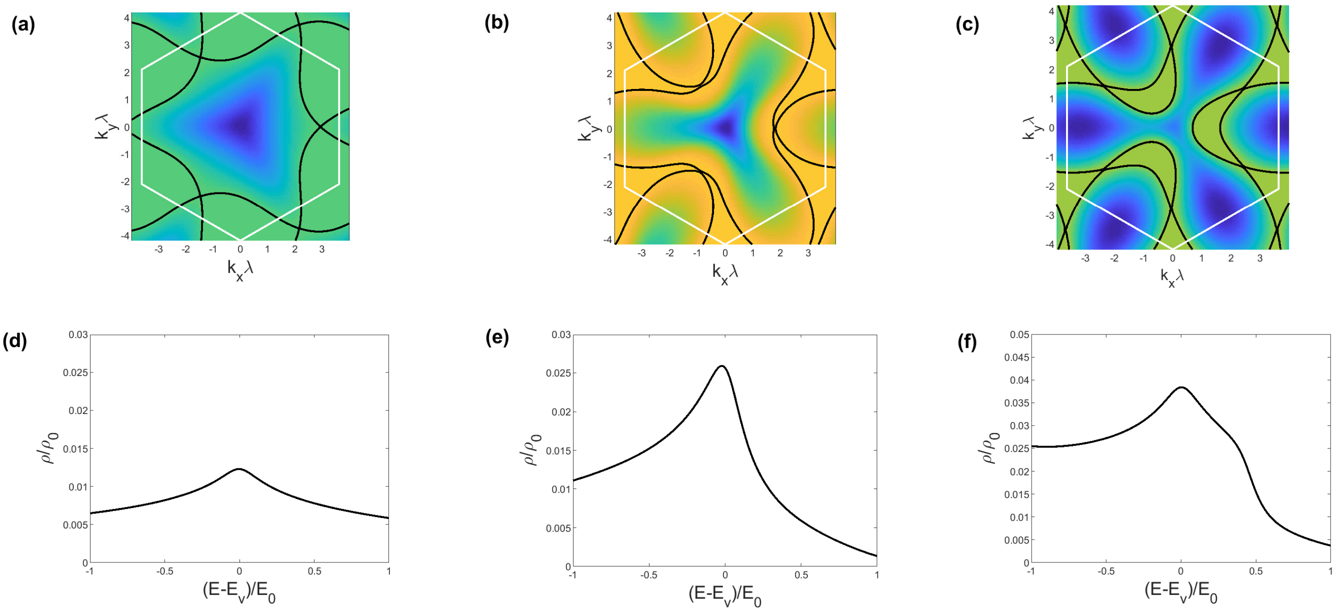

Supplementary Figure 4: (a,b,c) Energy contours and (d,e,f) total density of states calculated from continuum model. The coupling constants are $g' = 1.2g$ and (a,d) $g = 1$, (b,e) $g = 1.9$ and (c,f) $g = 2$. Here $E_{\rm v}$ is the energy of VHS, $E_0 = \alpha^3/\tilde{\gamma}^2, \rho_0 = \alpha^{-1}$, and $\alpha, \beta, \tilde{\gamma}$ are calculated from numerical derivatives of band structure at VHS. The broadening in DOS calculation is $\eta = 2.2 \times 10^{-3} E_0$.

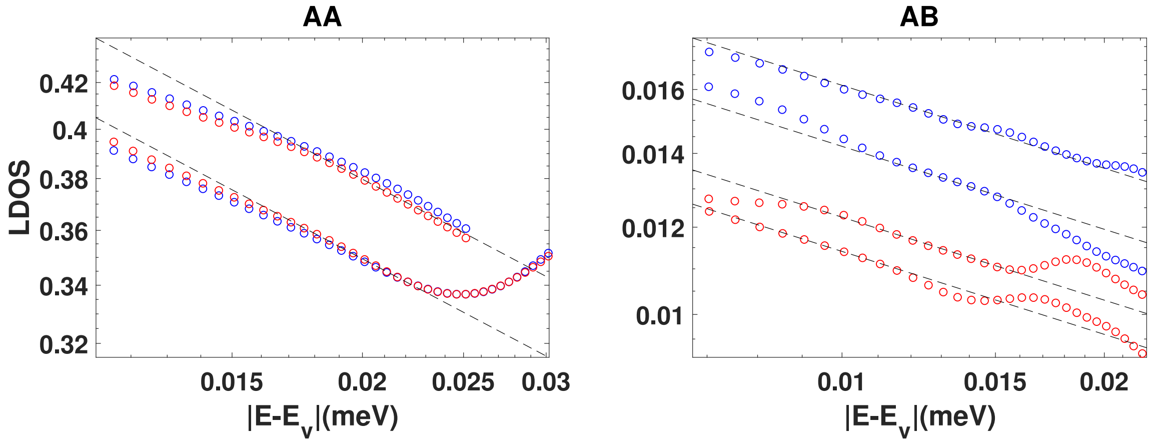

Supplementary Figure 5: Local density of states (LDOS) in AA and AB regions of twisted bilayer graphene at magic angle, where both LDOS and energy are plot in log scale.

We then put the ABC-TLG on top of hexagonal boron nitride (h-BN) and moiré superlattice is formed [11, 12]. We consider the non-twisting case and register the hexagon centers of ABC-TLG and h-BN. For simplicity we assume in the mini Brillouin zone (MBZ) of the superlattice, $\pm\boldsymbol{K}$ points of ABC-TLG and h-BN are folded to $\pm\boldsymbol{K}$ points of MBZ (Fig. 6c), and there exists emergent valley U(1) symmetry. With the substrate of h-BN, the symmetries that relate different layers are broken, and the remaining point group is C_{3v} generated by C_{3z} and M_x.

Denote the lattice constants of ABC-TLG and h-BN as a_G and a_{BN} respectively. For simplicity, we assume there exist two coprime positive integers m, n such that $ma_G = na_{BN} = L_{\rm M}$ is the superlattice constant. Denote $\delta = (a_G - a_{BN})/a_{BN}$ as the lattice constant mismatch, then moiré superlattice constant is $L_{\rm M} = a_G/|\delta|$.

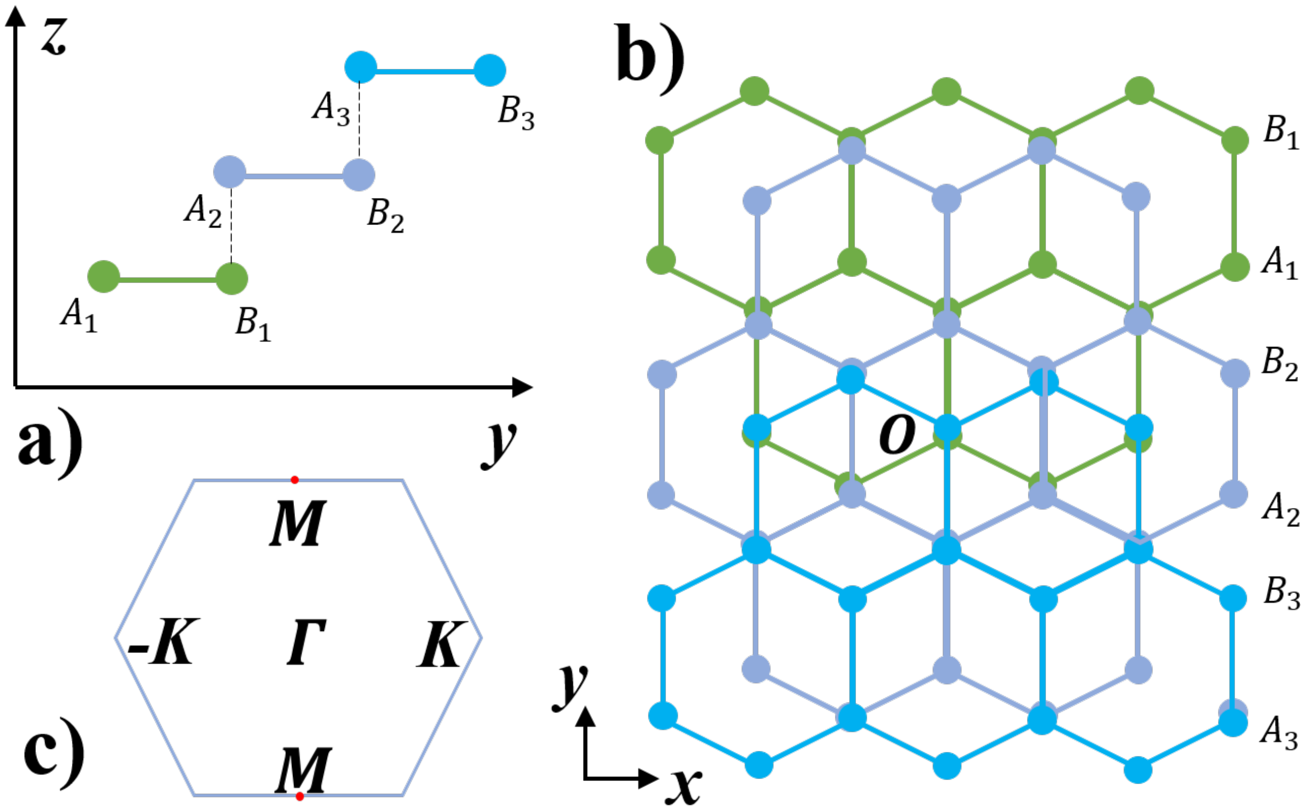

Supplementary Figure 6: Trilayer Graphene with ABC stacking, denoted as ABC-TLG. a) Side view. b) Top view. The origin O is chosen as the hexagon center of second layer, which is also the registered site of A_1 and B_3. c) Brillouin zone of b).

The substrate h-BN will create a periodic potential on the moiré scale

$$V_\xi(\boldsymbol{r}) = V_\xi \frac{1+\xi\sigma_z}{2} \sum_{j=0}^{2} \cos(\boldsymbol{G}_j \cdot \boldsymbol{r} + \phi_\xi) + V\sigma_z \tag{11}$$

where $\boldsymbol{G}_j = 4\pi/(\sqrt{3}L_{\mathrm{M}})(-\sin\frac{2\pi j}{3}, \cos\frac{2\pi j}{3})$, and $\xi = \pm$ denotes different alignments of TLG and h-BN.

A. High-order van Hove singularity

In this section we consider alignment scheme $\xi = -1$ where only B sublattice is affected by periodic potential of h-BN. The moiré parameters are

$$\delta = -0.017, \quad V_- = 12.09\mathrm{meV}, \quad \phi_- = -0.2591\pi. \tag{12}$$

Near electric field $V = 0.06$eV, the band structures of ABC-TLG on h-BN are as shown in Fig. 7. We focus on the valence bands denoted as Upper. Near K' point, we can expand the dispersion of the upper valence band as

$$E(\boldsymbol{p} - \boldsymbol{K}) = E_{\mathrm{v}} + \alpha p^2 + \kappa(p_x^3 - 3p_x p_y^2) \tag{13}$$

where the coefficients α and κ are functions of electric field V, and E_{v} is the energy of K' point. Near transition field $V_c = 0.06$eV, the corresponding Fermi contours are shown in Fig. 8. We find that when $V = V_c$, $\alpha = 0$, and K' point is a type-I high-order VHS where second order coefficients all vanish and three Fermi surfaces merge together.

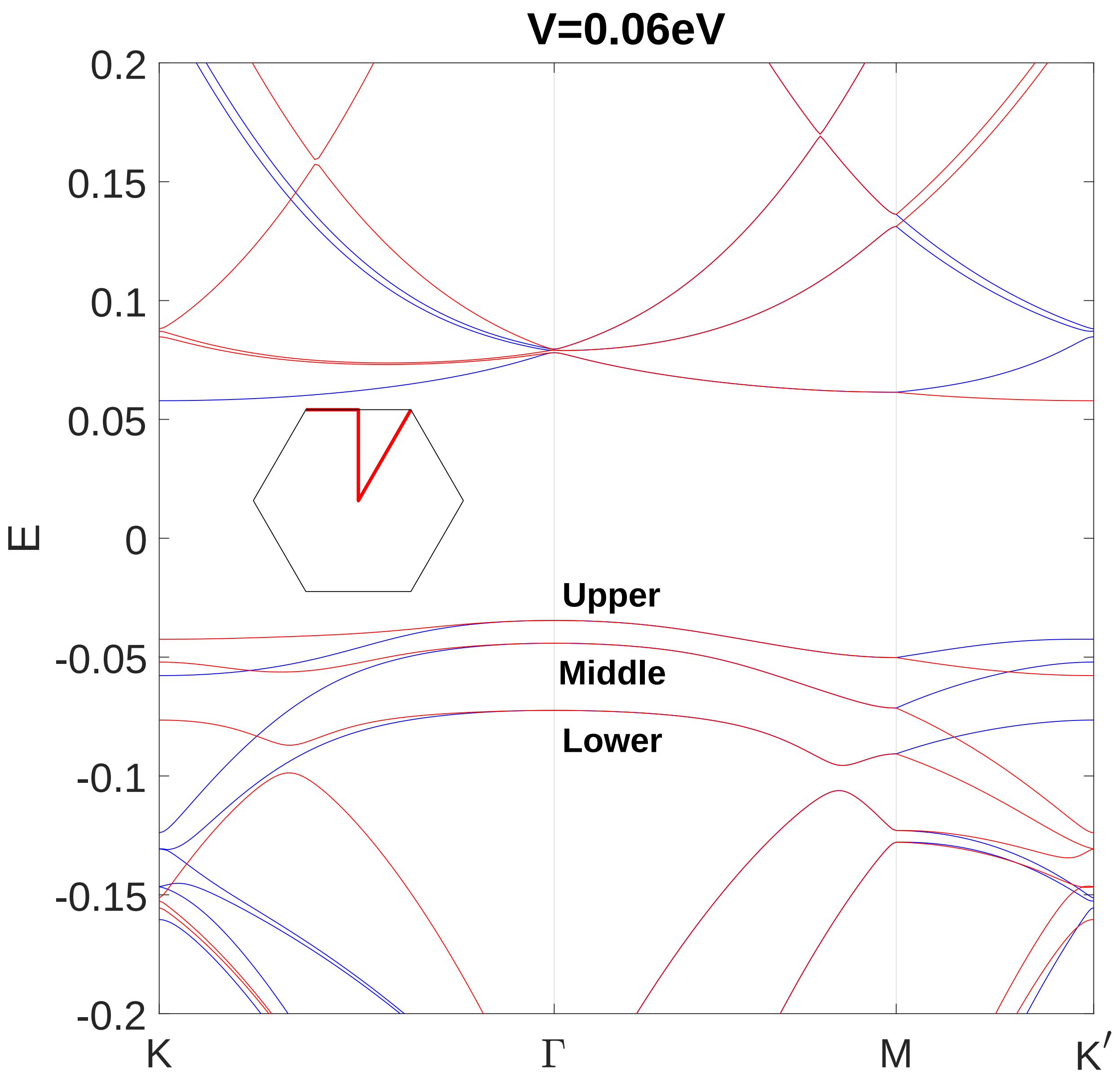

Supplementary Figure 7: Band structure of trilayer graphene on boron nitride (unit: eV) at a specific electric field strength $V = 0.06$eV with parameters in Eq. (12), where blue and red colors denote different valleys, and upper, middle and lower bands are specified.

When $V = V_c$, $\alpha = 0$, and the density of states near the type-I high-order VHS reads [16]

$$\rho(E) = C\kappa^{-\frac{2}{3}}|E - E_{\mathrm{v}}|^{-\frac{1}{3}}, \quad C = \frac{\Gamma(1/6)}{12\pi^{3/2}\Gamma(2/3)} = 0.06. \tag{14}$$

We find the DOS is power-law divergent and particle-hole symmetric.

The same topological transition and hence type-I high-order VHS can also be realized in the Lower valence band, either at K' point when $V = 0.11$eV, or at Γ point when $V = 0.14$eV.

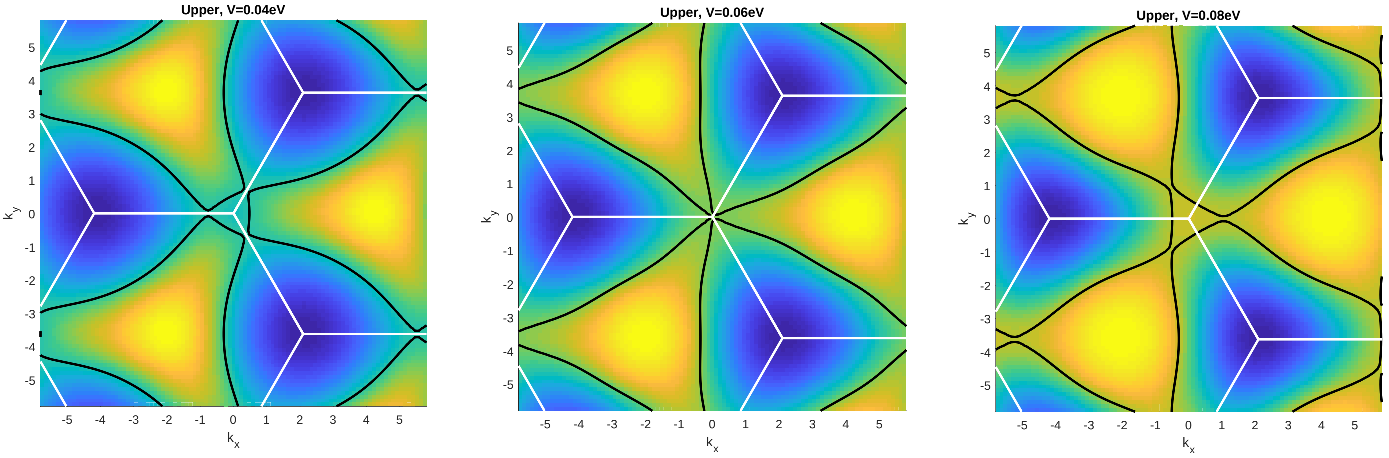

Supplementary Figure 8: Topological transition in trilayer graphene on boron nitride as electric field V changes in Eq. (11).

B. Effective tight-binding model

We introduce Wannier orbital $c_\tau(\boldsymbol{R})$ from valley τ at site $\boldsymbol{R}$, and they furnish the following representations of C_{3v} and valley U(1) group

$$U(C_{3z})c_\tau(\boldsymbol{R}) = c_\tau(C_{3z}\boldsymbol{R}), \quad U(M_x)c_\tau(\boldsymbol{R}) = c_{-\tau}(M_x\boldsymbol{R}), \quad U(\phi)c_\tau(\boldsymbol{R}) = e^{i\phi}c_\tau(\boldsymbol{R}) \tag{15}$$

which is essentially projective

$$U(g_1)U(g_2) = U(\phi)U(g_1g_2), \quad \forall g_1, g_2 \in C_{3v}. \tag{16}$$

The emergent symmetry group of TLG-hBN system is hence ($\pm$ denote different valleys)

$$\mathcal{G} = C_{3v} \ltimes \mathrm{U}(1) \times \mathrm{SU}(2)_+ \times \mathrm{SU}(2)_- \times \mathcal{T}. \tag{17}$$

Since there are no Dirac points in such system, we can construct the effective tight-binding model on a triangular lattice in terms of valley basis $c = (c_+, c_-)$

$$H_{tb} = \sum_{ij} t_{ij} c_i^\dagger e^{i\phi_{ij}\sigma_z} c_j + h.c. \tag{18}$$

Due to M_x symmetry, $\phi_{ij} = 0$ unless the bond is along three directions $\hat{\boldsymbol{e}}_{ij} = C_{3z}^n \hat{\boldsymbol{x}}$ for $n = 0, 1, 2$.

For moiré parameters in alignment scheme $\xi = +1$ where only A sublattice is affected by periodic potential of h-BN

$$\delta = -0.017, \quad V_+ = -14.88\text{meV}, \quad \phi_+ = 0.2788\pi, \quad V = 70\text{meV}, \tag{19}$$

we use the following tight-binding model to fit the two trivial bands in the continuum model spectrum

$$H_{tb} = \sum_{\langle ij \rangle} t_1 c_i^\dagger e^{i\phi\sigma_z} c_j + \sum_{\langle\langle ij \rangle\rangle} t_2 c_i^\dagger c_j + h.c. \tag{20}$$

and the corresponding fitting parameters of the lowest conduction bands are

$$t_1 = 1.20\text{meV}, \quad \phi = -0.48\pi, \quad t_2 = 0.45\text{meV}. \tag{21}$$

Since ϕ is close to $\pi/2$, the nearest neighbor term mostly contributes to the warping effect. Fittings both along specific path and in the entire MBZ are shown in Figs. 9 and 10 respectively.

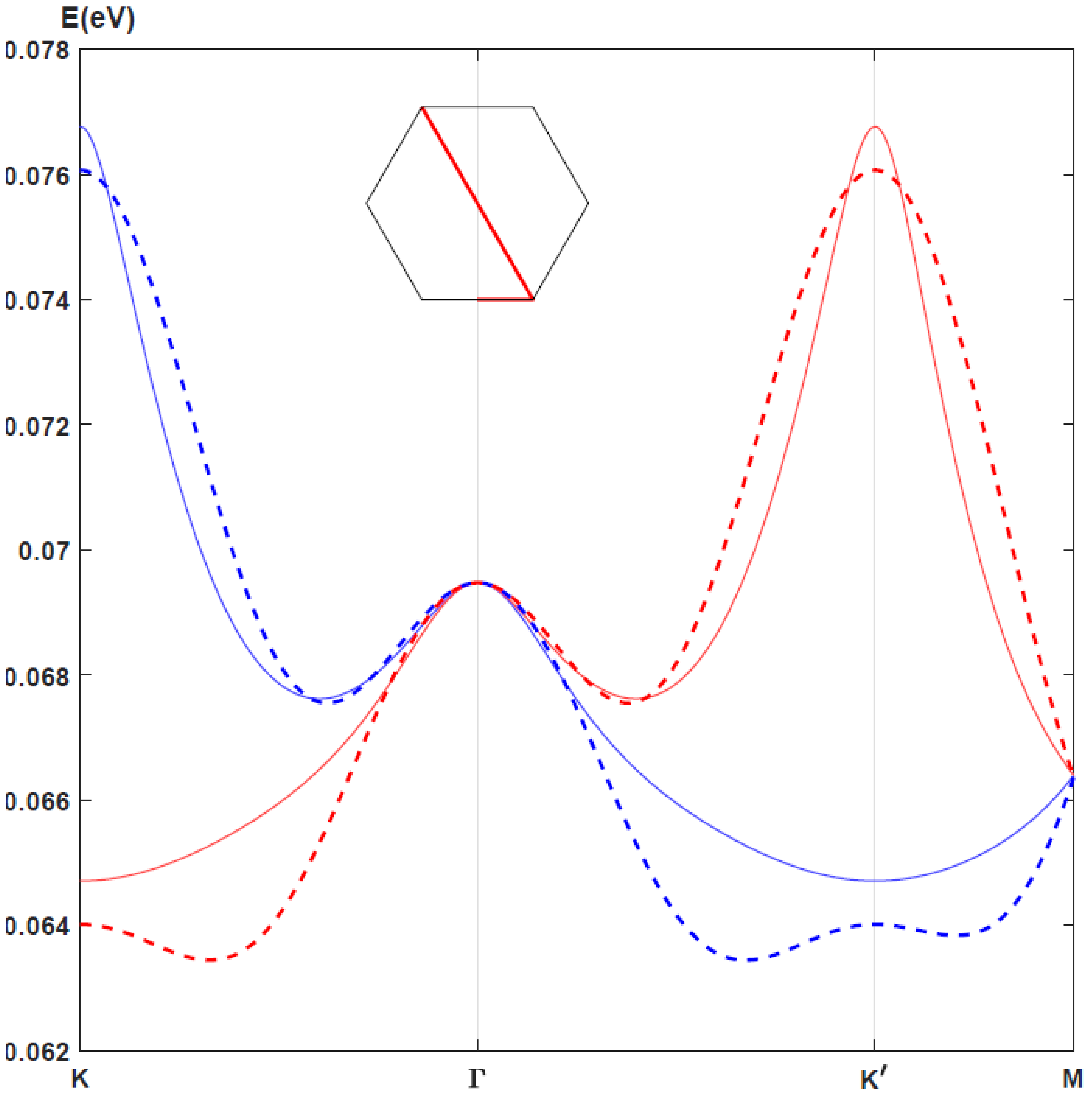

Supplementary Figure 9: Continuum model (solid) and tight-binding (dashed) band structures of trilayer graphene on boron nitride, along the path indicated by red lines in the Brilluoin zone. Red and blue denote different valleys. Parameters of continuum model are given in Eq. (19), and parameters of tight-binding model are given in Eq. (21).

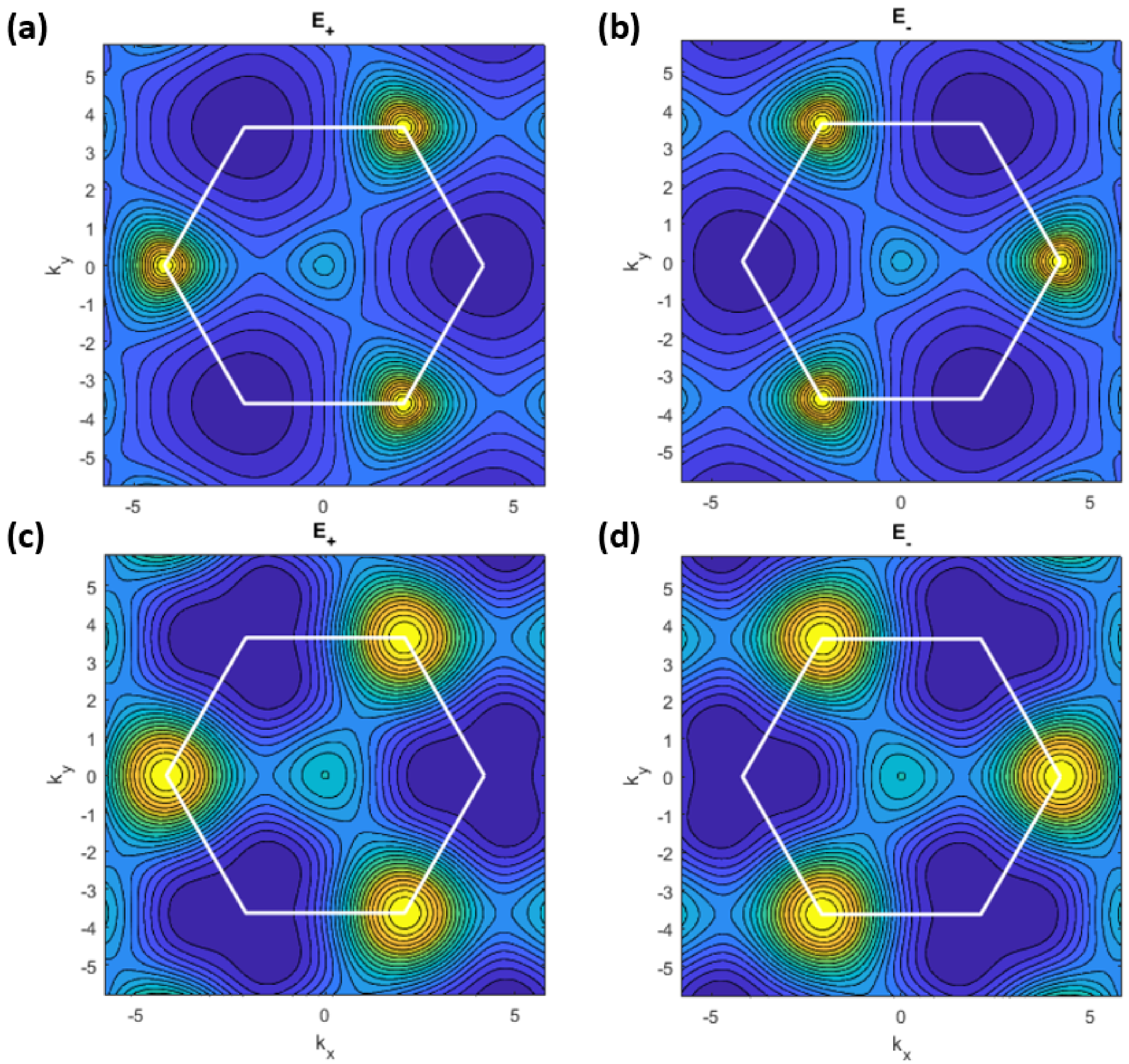

Supplementary Figure 10: Energy contours calculated from continuum model (a and b) and fitting of tight-binding model (c and d) for different valleys (a, c for positive valley and b, d for negative valley). Parameters of continuum model are given in Eq. (19), and parameters of tight-binding model are given in Eq. (21).

SUPPLEMENTARY REFERENCES

[1] A. Kerelsky, L. McGilly, D. M. Kennes, L. Xian, M. Yankowitz, S. Chen, K. Watanabe, T. Taniguchi, J. Hone, C. Dean, A. Rubio, and A. N. Pasupathy, *Maximized electron interactions at the magic angle in twisted bilayer graphene*, Nature **572**, 95-100 (2019).

[2] M. Koshino, N. F. Q. Yuan, T. Koretsune, M. Ochi, K. Kuroki, and L. Fu, *Maximally Localized Wannier Orbitals and the Extended Hubbard Model for Twisted Bilayer Graphene*, Phys. Rev. X **8**, 031087 (2018).

[3] M. Yankowitz, S. Chen, H. Polshyn, K. Watanabe, T. Taniguchi, D. Graf, A. F. Young, and C. R. Dean, *Tuning superconductivity in twisted bilayer graphene*, Science 10.1126/science.aav1910 (2019).

[4] N. N. T. Nam and M. Koshino, *Lattice relaxation and energy band modulation in twisted bilayer graphene*, Phys. Rev. B **96**, 075311 (2017).

[5] S. Carr, S. Fang, Z. Zhu, E. Kaxiras, *Minimal model for low-energy electronic states of twisted bilayer graphene*, Phys. Rev. Research **1**, 013001 (2019).

[6] Zhen Bi, Noah F. Q. Yuan, Liang Fu, *Designing Flat Band by Strain*, Phys. Rev. B **100**, 035448 (2019).

[7] Kasra Hejazi, Chunxiao Liu, Hassan Shapourian, Xiao Chen, and Leon Balents, *Multiple topological transitions in twisted bilayer graphene near the first magic angle*, Phys. Rev. B **99**, 035111 (2019).

[8] D. C. Elias, R. V. Gorbachev, A. S. Mayorov, S. V. Morozov, A. A. Zhukov, P. Blake, L. A. Ponomarenko, I. V. Grigorieva, K. S. Novoselov, F. Guinea, and A. K. Geim, *Dirac cones reshaped by interaction effects in suspended graphene*, Nat. Phys. **7**, 701 (2011).

[9] G. L. Yu, R. Jalil, B. Belle, A. S. Mayorov, P. Blake, F. Schedin, S. V. Morozov, L. A. Ponomarenko, F. Chiappini, S. Wiedmann, U. Zeitler, M. I. Katsnelson, A. K. Geim, K. S. Novoselov, and D. C. Elias, *Interaction phenomena in graphene seen through quantum capacitance*, Proc. Natl. Acad. Sci. **110**, 3282 (2013).

[10] T. Stauber, P. Parida, M. Trushin, M.V. Ulybyshev, D.L. Boyda, and J. Schliemann, *Interacting Electrons in Graphene: Fermi Velocity Renormalization and Optical Response*, Phys. Rev. Lett. **118**, 266801 (2017).

[11] Guorui Chen, Lili Jiang, Shuang Wu, Bosai Lyu, Hongyuan Li, Bheema Lingam Chittari, Kenji Watanabe, Takashi Taniguchi, Zhiwen Shi, Jeil Jung, Yuanbo Zhang and Feng Wang , *Evidence of a gate-tunable Mott insulator in a trilayer graphene moir superlattice*, Nat. Phys. **15**, 237 (2019).

[12] Guorui Chen, Aaron L. Sharpe, Patrick Gallagher, Ilan T. Rosen, Eli Fox, Lili Jiang, Bosai Lyu, Hongyuan Li, Kenji Watanabe, Takashi Taniguchi, Jeil Jung, Zhiwen Shi, David Goldhaber-Gordon, Yuanbo Zhang, Feng Wang, *Signatures of Gate-Tunable Superconductivity in Trilayer Graphene/Boron Nitride Moir Superlattice*, Nature **572**, 215-219 (2019).

[13] Guo-Yi Zhu, Tao Xiang, Guang-Ming Zhang, *Inter-valley spiral order in the Mott insulating state of a heterostructure of trilayer graphene-boron nitride*, Science Bulletin **63**, 63 (2018).

[14] Bheema Lingam Chittari, Guorui Chen, Yuanbo Zhang, Feng Wang, and Jeil Jung, *Gate-Tunable Topological Flat Bands in Trilayer Graphene Boron-Nitride Moir Superlattices*, Phys. Rev. Lett. **122**, 016401 (2019).

[15] Ya-Hui Zhang, Dan Mao, Yuan Cao, Pablo Jarillo-Herrero, and T. Senthil, *Nearly flat Chern bands in moir superlattices*, Phys. Rev. B **99**, 075127 (2019).

[16] A. Shtyk, G. Goldstein, and C. Chamon, *Electrons at the monkey saddle: A multicritical Lifshitz point*, Phys. Rev. B **95**, 035137 (2017).
